# β-Lactam Dosage Regimens in Septic Patients with Augmented Renal Clearance

**DOI:** 10.1128/AAC.02534-17

**Published:** 2018-08-27

**Authors:** Alexandra Jacobs, Fabio Silvio Taccone, Jason A. Roberts, Frédérique Jacobs, Frederic Cotton, Fleur Wolff, Jacques Creteur, Jean-Louis Vincent, Maya Hites

**Affiliations:** a Department of Intensive Care, CUB-Erasme, Université Libre de Bruxelles, Brussels, Belgium; b Department of Infectious Diseases, CUB-Erasme, Université Libre de Bruxelles, Brussels, Belgium; c University of Queensland Centre for Clinical Research, Royal Brisbane and Women's Hospital, Brisbane, Queensland, Australia; d School of Pharmacy, Centre for Translational Anti-infective Pharmacodynamics, University of Queensland, Woolloongabba, Queensland, Australia; e Department of Intensive Care Medicine, Royal Brisbane and Women's Hospital, Brisbane, Queensland, Australia; f Department of Pharmacy, Brisbane and Women's Hospital, Brisbane, Queensland, Australia; g Department of Clinical Biochemistry, CUB-Erasme, Université Libre de Bruxelles, Brussels, Belgium

**Keywords:** creatinine clearance, meropenem, ceftazidime, cefepime, piperacillin-tazobactam, critically ill, pharmacokinetics

## Abstract

Augmented renal clearance is commonly observed in septic patients and may result in insufficient β-lactam serum concentrations. The aims of this study were to evaluate potential correlations between drug concentrations or total body clearance of β-lactam antibiotics and measured creatinine clearance and to quantify the need for drug dosage adjustments in septic patients with different levels of augmented renal clearance. We reviewed 256 antibiotic measurements (512 drug concentrations) from a cohort of 215 critically ill patients who had a measured creatinine clearance of ≥120 ml/min and who received therapeutic drug monitoring of meropenem, cefepime, ceftazidime, or piperacillin from October 2009 until December 2014 at Erasme Hospital. Population pharmacokinetic (PK) analysis of the data was performed using the Pmetrics software package for R. Fifty-five percent of drug concentrations showed insufficient β-lactam serum concentrations to treat infections due to Pseudomonas aeruginosa. There were significant, yet weak, correlations between measured creatinine clearance and trough concentrations of meropenem (*r* = −0.21, *P* = 0.01), trough concentrations of piperacillin (*r* = −0.28, *P* = 0.0071), concentrations at 50% of the dosage interval (*r* = −0.41, *P* < 0.0001), and total body clearance of piperacillin (*r* = 0.39, *P* = 0.0002). Measured creatinine clearance adequately explained changes in drug concentrations in population pharmacokinetic models for cefepime, ceftazidime, and meropenem but not for piperacillin. Therefore, specific PK modeling can predict certain β-lactam concentrations based on renal function but not on absolute values of measured creatinine clearance, easily available for clinicians. Currently, routine therapeutic drug monitoring is required to adjust daily regimens in critically ill patients receiving standard dosing regimens.

## Abstract (precis)

Augmented renal clearance is commonly observed in septic patients and may result in insufficient β-lactam serum concentrations. The aims of this study were to evaluate potential correlations between drug concentrations or total body clearance of β-lactam antibiotics and measured creatinine clearance and to quantify the need for drug dosage adjustments in septic patients with different levels of augmented renal clearance.

## INTRODUCTION

Augmented renal clearance (ARC) refers to the enhanced elimination of solutes by the kidneys, and it is common in critically ill patients (1). Reported incidence rates vary significantly (16 to 100%) depending on the patient population studied and the criteria applied (2–8). ARC is usually defined as a creatinine clearance (CL_CR_) of >130 ml/min/1.73 m^2^ (1), calculated from either an 8- or 24-h urine collection (5, 9, 10).

In the septic patient, ARC is typically due to an increased cardiac output, resulting in an increased renal blood flow, and therefore an increased glomerular filtration rate. An important consequence of ARC is the increased risk of suboptimal achievement of pharmacokinetic/pharmacodynamic (PK/PD) targets for hydrophilic antibiotics, such as β-lactams, when standard drug regimens are administered (1). Suboptimal PK/PD target achievement may lead to emergence of resistance and/or therapeutic failure. Studies evaluating outcomes in critically ill patients with ARC and receiving β-lactam antibiotics are scarce and have shown conflicting results (8, 11). However, several studies, including an international, multicentric study including 68 intensive care units (ICUs), have demonstrated an association between suboptimal achievement of PK/PD targets and poor outcome in the general critically ill patient population (8, 12–14).

Only four studies have evaluated the relationship between levels of CL_CR_ and β-lactam serum concentrations among patients with ARC. In all four, as CL_CR_ increased, the probability of PK/PD target attainment of piperacillin-tazobactam (TZP), meropenem (MEM), cefepime (FEP), or ceftazidime (CAZ) decreased significantly (11, 15–17). However, the populations studied were small (from 48 to 61 patients), patients without ARC were included, most data came from patients receiving TZP (15, 16), and CL_CR_ was estimated with the Cockcroft-Gault equation (18), an unreliable method to assess renal function in critically ill patients (5, 9). Further analyses are needed to help adjust drug regimens according to the degree of increase in CL_CR_, similar to the downward dose adjustments in patients with renal failure (19).

Thus, the aims of the present study were (i) to characterize the PKs of several broad-spectrum β-lactams, (ii) to evaluate a potential correlation between the drug concentrations or total body clearance (CL) of β-lactams with measured CL_CR_ (mCL_CR_), and (iii) to quantify the need for dose adjustment in critically ill patients with different levels of ARC.

## RESULTS

### Study population.

We evaluated 256 therapeutic drug monitorings (TDMs) in 215 patients, corresponding to 256 measurements of β-lactam serum concentrations before (*T*_0_) and 256 measurements 2 h after the initiation of the β-lactam infusion (*T*_2_). The TDMs consisted of the following: 11 for FEP (4%), 11 for CAZ (4%), 89 for piperacillin (PIP) (35%), and 145 for MEM (67%). Only 10 of the 512 measured serum concentrations were below the limits of quantification (<1 mg/liter), all measured at *T*_0_. The characteristics of the study population are shown in Table 1. Patients were predominantly male, with a median age of 56 years, a median acute physiology and chronic health evaluation (APACHE) II score of 19 on ICU admission, and a median sequential organ failure assessment (SOFA) score of 6 on the day of TDM (20, 21). The most frequent site of infection was the respiratory tract, and the most frequently encountered pathogens were Enterobacteriaceae spp. (43%) (Table 2).

**TABLE 1:** Demographic, biological and clinical characteristics of all patients

Patient characteristic^*a*^	Value for the group (*n* = 215)^*b*^
Demographics	
Age (yr)	57 (42–65)
No. of males (%)	152 (71)
No. of female (%)	63 (29)
Body wt (kg)	73 (62–84)
No. of medical admissions (%)	148 (69)
Comorbidities (no. of patients [%])	
Heart disease	48 (22)
COPD/asthma	47 (22)
Diabetes	45 (21)
Immunosuppression	32 (15)
Cancer	31 (14)
Liver cirrhosis	17 (8)
Biological data	
Serum creatinine (mg/dl)	0.6 (0.2–1.0)
mCL_CR_ (ml/min)	179 (148–233)
Clinical data	
APACHE II score on ICU admission	19 (14–24)
SOFA score on day of TDM	6 (1–11)
ICU LOS (days)	12 (7–23)
ICU 30-day mortality (no. of patients [%])	35 (16)

a COPD, chronic obstructive pulmonary disease; mCL_CR_, measured creatinine clearance; APACHE, acute physiology and chronic health evaluation; SOFA, sequential organ failure assessment; ICU, intensive care unit; LOS, length of stay.

b Values are medians (IQR) unless otherwise noted.

**TABLE 2:** Characteristics of infections and identified pathogens in the study cohort

Infection parameter	No. (%)
Site	
Respiratory	138 (54)
Abdomen	49 (19)
Primary bacteremia	17 (6)
Central nervous system	15 (6)
Skin	9 (3)
Catheter	5 (2)
Urinary tract	5 (2)
Mediastinitis	5 (2)
Unknown	13 (5)
Pathogen(s)	
Enterobacteriaceae spp.	110 (43)
Pseudomonas aeruginosa	34 (13)
Staphylococcus aureus	22 (9)
Enterococcus spp.	9 (3)
Staphylococcus epidermidis	7 (3)
Acinetobacter spp.	4 (1)
Other	33 (3)
Unidentified	37 (14)
Patients with positive blood cultures	50 (20)

### Creatinine clearance and insufficient drug concentrations.

**FIG 1:**
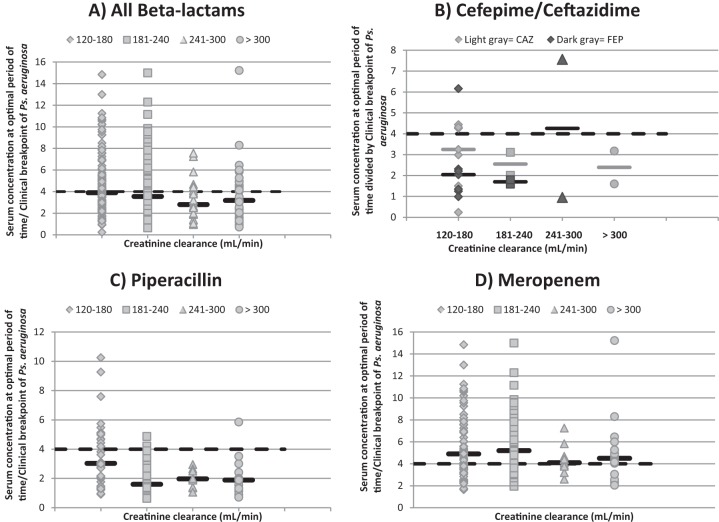
Serum concentrations of β-lactams (at optimal period of time divided by clinical breakpoints of Pseudomonas aeruginosa), in function of quartiles of mCL_CR_, as indicated. Solid horizontal black lines, median serum concentrations; dashed horizontal black lines, pharmacodynamic target of 4× MIC of the clinical breakpoints for Pseudomonas aeruginosa. Serum concentration at optimal period of time is the serum concentration at 40%, 50%, or 70% of the dosage interval of MEM, TZP, or FEP/CAZ, respectively.

The median mCL_CR_ of the study cohort at the time of TDM was 178 ml/min. The distribution of the TDM values relative to mCL_CR_ is shown in Table 3. The majority of TDMs were performed in patients with mCL_CR_s varying from 120 to 180 ml/min. Insufficient drug concentrations to treat infections due to Pseudomonas aeruginosa were observed in 141 of these 256 TDMs (55%); the proportion of patients with insufficient concentrations was significantly greater for FEP, CAZ, and PIP than for MEM (82%, 73%, 79% and 37%, respectively; *P* < 0.001). As mCL_CR_ quartiles increased to an mCL_CR_ of 240 to 300 ml/min, the proportion of patients with insufficient serum concentrations of all β-lactams, of PIP, and of MEM increased (from 49% to 71%, from 58% to 100%, and from 38% to 45%, respectively), as illustrated in Fig. 1. For TDMs of FEP and CAZ, no correlation was observed between proportions of insufficient serum concentrations and the different mCL_CR_ levels; however, only eight TDMs were available for evaluation in patients with mCL_CR_s of >180 ml/min.

**TABLE 3:** Distribution of therapeutic drug monitorings as a function of measured creatinine clearance intervals

Antibiotic(s)^*a*^	No. (%) of TDMs at CL_CR_ of:^*b*^			
	120–180 ml/min	181–240 ml/min	241–300 ml/min	>300 ml/min
All	130 (51)	67 (26)	24 (9)	35 (14)
FEP	7 (32)	2 (9)	2 (9)	0 (0)
CAZ	7 (32)	2 (9)	0 (0)	2 (9)
PIP	43 (48)	22 (25)	11 (12)	13 (15)
MEM	73 (50)	41 (28)	11 (8)	20 (14)

a FEP, cefepime; CAZ, ceftazidime; PIP, piperacillin; MEM, meropenem.

b Creatinine clearance (CL_CR_) obtained from 24-h urine collections. TDMs, therapeutic drug monitorings.

Because Enterobacteriaceae spp. were the most frequently documented pathogens in this study, adequacy of drug concentrations to treat infections due to these pathogens was also evaluated. Insufficient drug concentrations to treat infections due to Enterobacteriaceae spp. were observed in 93 of the 256 TDMs (36%): 1/11 (10%) for FEP, 0/11 (0%) for CAZ, 38/89 (43%) for PIP, and 54/145 (37%) for MEM. Once again, as mCL_CR_ quartiles increased to mCL_CR_s of 241 to 300 ml/min, the proportion of patients with insufficient serum concentrations of all β-lactams, of PIP, and of MEM increased (from 31% to 50%, from 26% to 64%, and from 38% to 45%, respectively).

### Creatinine clearance and drug PKs.

**FIG 2:**
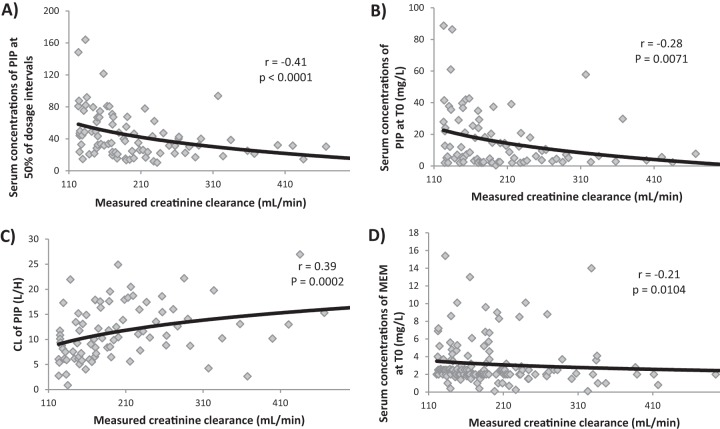
Logarithmic correlations between mCL_CR_ and different parameters. (A) Serum concentrations of PIP at 50% of the dosage interval and mCL_CR_. (B) Serum trough concentrations of PIP at *T*_0_ and mCL_CR_ (ml/min). (C) Total body clearance (CL) of PIP and mCL_CR_. (D) Serum trough concentrations of MEM at *T*_0_ and mCL_CR_. PIP, piperacillin; mCL_CR_, measured creatinine clearance; MEM, meropenem.

Drug PKs are provided in Table 4. No significant correlations were observed between mCL_CR_ and the studied parameters for FEP and CAZ (data not shown). For PIP, we observed a significant, but weak, logarithmic correlation between mCL_CR_ and all the studied parameters, except for half-life (*t*_1/2_): between mCL_CR_ and trough concentrations (*T*_0_s) of PIP (*r* = −0.28, *P* = 0.0071), PIP at 50% of the dosage interval (*r* = −0.41, *P* < 0.0001), and CL of PIP (*r* = 0.39, *P* = 0.0002) (Fig. 2). Finally, for MEM we also found a significant, but weak, logarithmic correlation between mCL_CR_ and *T*_0_, as shown in Fig. 2 (*r* = −0.21, *P* = 0.01), but not for the other parameters (data not shown).

**TABLE 4:** Pharmacokinetics, elimination constant, and half-life of the different β-lactam antibiotics and measured creatinine clearance

Parameter^*a*^	Median value for the parameter (IQR)^*b*^			
	FEP (*n* = 11)	CAZ (*n* = 11)	PIP (*n* = 89)	MEM (*n* = 145)
Serum concn at CT (mg/liter)	12.7 (10.0–18.5)	24.9 (12.8–34.1)	35.2 (23.2–56.2)	9.1 (6.9–12.9)
Serum concn at *T*_0_ (mg/liter)	6.9 (5.0–10.9)	16.0 (5.7–20.7)	7.0 (2.8–22.0)	3.3 (2.0–3.2)
Serum concn at *T*_2_ (mg/liter)	40.7 (35.4–48.3)	42.0 (31.8–59.5)	65.0 (45.5–83.7)	12.0 (8.6–17.5)
%*T* >4× MIC	34.5 (28.1–42.7)	53.4 (34.8–73.8)	34.2 (21.5–44.5)	46.9 (33.0–60.7)
mCL_CR_ (ml/min)	155 (140–199)	173 (155–226)	186 (149–249)	179 (146–231)
*k_e_* (h^−1^)	0.3 (0.2–0.4)	0.2 (0.2–0.3)	0.5 (0.3–0.7)	0.3 (0.2–0.3)
Half-life (h)	2.5 (2.0–3.0)	3.3 (2.2–4.2)	2.2 (1.0–1.4)	2.7 (2.2–3.4)

a CT, serum concentration at optimal period of time (i.e., at 40%, 50%, or 70% of the dosage interval of MEM, TZP, or FEP/CAZ, respectively); *T*_0_, trough; *T*_2_, 2 h after onset of the β-lactam infusion; %*T* >4× MIC, percentage of time during which the antibiotic remains above 4× MIC; mCL_CR_, measured creatinine clearance; *k_e_*, elimination constant.

b FEP, cefepime; CAZ, ceftazidime; PIP, piperacillin; MEM, meropenem; *n*, number of TDMs.

### Population PK analysis.

Each TDM contributed to the analysis. The best models were one-compartment linear models with zero-order input. Other models could not be supported. Between-subject variability was tested during model building for both total body clearance (CL) and volume of distribution (*V*). The final −2× log likelihood (−2LL) values for the base models for FEP, CAZ, PIP, and MEM were 168.5, 126.6, 1,274, and 1,064, respectively. Population PK parameter estimates are provided in Table 5. There was significant between-subject variability of *V* and CL for all drugs. The values of between-subject variability with mCL_CR_ (normalized to 130 ml/min) for FEP, CAZ, PIP, and MEM were 24.7%, −1.1%, 5.7%, and −13.8%, respectively (negative values indicate increased variability present). mCL_CR_ improved the models for FEP, CAZ, and MEM and could be retained in the final model. The goodness-of-fit plots for the final models are displayed in Fig. S1 in the supplemental material with *r*^2^ correlations between observed and posterior predicted concentrations of the final models of 0.963 (mCL_CR_ covariate supported), 0.994 (mCL_CR_ covariate supported), 0.994 (mCL_CR_ covariate not supported), and 0.996 (mCL_CR_ covariate supported), respectively, as well as little systematic bias evident for any drugs. All other visual predictive checks (VPC) were acceptable and confirmed the goodness of fit of the final model. We found that mCL_CR_ could adequately explain changes in drug CL or concentrations for FEP, CAZ, and MEM but not for PIP.

**TABLE 5:** Population PK parameter estimates of the different β-lactam antibiotics from the one-compartment model

Drug (*n*)^*a*^	*V*^*b*^				CL^*c*^			
	Mean (liters)	CV (%)	Variance (liters)	Median (liters)	Mean (liters/h)	CV (%)	Variance (liters/h)	Median (liters/h)
FEP (11)	47.8	15.7	56.5	50.0	18.8	106	401.2	10.1
CAZ (11)	63.4	44.4	790.9	47.1	13.1	68.9	81.1	10.0
PIP (89)	56.3	30.4	293.2	49.6	26.8	52.4	197.1	25.9
MEM (145)	77.2	41.4	1,020.3	76.0	15.0	56.7	72.2	13.5

a FEP, cefepime; CAZ, ceftazidime; PIP, piperacillin; MEM, meropenem; *n*, number of TDMs.

b *V*, volume of distribution in the central compartment; CV, coefficient of variation.

c CL, total body clearance.

## DISCUSSION

In this study, we found that β-lactam serum concentrations were insufficient to treat infections due to Pseudomonas aeruginosa in the majority of septic patients with ARC. This was particularly significant for the patients with very high mCL_CR_ (>180 ml/min) values. Linear and/or logarithmic correlations between mCL_CR_ and drug concentrations and between mCL_CR_ and CL of β-lactams were weak or absent. Specific population PK modeling could predict concentrations of FEP, CAZ, and MEM (but not of PIP) based on renal function but not based on absolute values of mCL_CR_. Therefore, a simple upward dose adjustment of these β-lactam antibiotics cannot be proposed on the basis of only mCL_CR_. These results highlight the complexity of drug PKs in septic patients.

The majority of TDMs were performed in patients with an mCL_CR_ between 120 and 180 ml/min. In agreement with other studies on ARC, patients were young (6, 8, 22, 23) and predominantly male (8), with low plasma creatinine concentrations and relatively low severity-of-illness scores (6, 8, 15).

There is currently no consensus concerning the optimal PD targets for β-lactams in infected, critically ill patients. Indeed, few papers on the PKs of β-lactams in critically ill patients report MIC data or clinical outcome (24). Therefore, the clinical efficacy of different PD targets has not been robustly assessed. Nevertheless, we aimed for a PD target of >4× the MIC of the pathogen at the end of the optimal period of time (CT), corresponding to 70% of the dose interval for FEP/CAZ, 50% for PIP, and 40% for MEM, based on results from *in vitro* (25–27), animal *in vivo* (28), and clinical studies. Indeed, microbiological success has been predicted in patients with Gram-negative infections when FEP concentrations were maintained at 4× to 6× the MIC of the infecting pathogen (19). Another study has shown that unbound MEM trough serum concentrations at 5× the MIC of the infecting pathogen for patients with lower respiratory infections was predictive of microbiological and clinical success (12).

Antimicrobial treatment of sepsis is often initiated empirically when pathogens and MICs are unknown. We therefore aimed to treat infections due to Pseudomonas aeruginosa, a pathogen frequently responsible for infections in the ICU setting and associated with high mortality rates (29). However, we found that the most frequently documented pathogens in our study were Enterobacteriaceae spp. Although adequacy of drug concentrations was better in the treatment of infections due to Enterobacteriaceae spp. instead of those due to Pseudomonas aeruginosa, one out of three patients still had insufficient β-lactam serum concentrations to attain PK/PD targets.

We report, for the first time, a significantly greater proportion of insufficient concentrations of FEP, CAZ PIP, than of MEM for treatment of infections due to Pseudomonas spp. in patients with ARC. Indeed, in a prospective PK study, Carlier et al. reported that in 60 critically ill patients with apparently normal renal function (43 patients received TZP, and only 17 received MEM), no significant differences in pharmacodynamic target attainment were observed between antibiotics (15). In another study, De Waele et al. prospectively randomized critically ill patients with apparent normal kidney function to receive TDM-guided or standard therapy (MEM or TZP); baseline PK/PD target attainment for the entire cohort was similar for TZP and MEM (20/28, or 71.4%, and 6/13, or 46.2%, respectively; *P* = 0.12) (30). Huttner et al. also performed a prospective observational PK study in 100 critically ill patients with CL_CR_s of >60 ml/min who received MEM, TZP, FEP, or imipenem/cilastatin. Subtherapeutic concentrations of MEM and PIP were observed in 90% and 61% of trough samples, respectively, but ARC was present in 9/11 patients who received MEM and in only 21/33 patients who received TZP (11). In all three of these studies, fewer patients received MEM than TZP, not all patients had ARC, and CL_CR_ was estimated using the Cockcroft-Gault equation, yielding results that often differ significantly from measured values (5, 9). Whether our findings are due to drug characteristics or to the choice in target concentrations and whether this would translate into a more “effective” (i.e., less risk of underdosing) therapy with MEM than with TZP in critically ill patients need to be further evaluated in other studies.

In view of the large number of patients identified in this study with insufficient serum concentrations to attain PK/PD targets, optimization of treatment must be considered. Because β-lactam antibiotics are time dependent and because the majority have a short half-life, prolonged or continuous infusions of these drugs may theoretically improve PK/PD target attainment in cases of ARC by ensuring that serum antibiotic concentrations remain above the MIC of the infecting pathogen during a longer period of time (1). However, even when prolonged infusions of standard total daily doses of β-lactam antibiotics are administered in these circumstances, PK/PD attainment may still remain suboptimal (15). Hence, an increased dosage may be necessary.

Unfortunately, we are not yet able to provide a potential drug regimen adjustment based only on mCL_CR_. Indeed, only weak correlations were observed between mCL_CR_ and proportions of insufficient β-lactams, between mCL_CR_ and PIP serum concentrations, between mCL_CR_ and CL of PIP, and between mCL_CR_ and *T*_0_ of MEM. For FEP and CAZ, no correlations were found between increasing values of ARC, proportion of insufficient drug levels, drug levels at different time points, or CL. Finally, for all β-lactams studied, no correlations were found between mCL_CR_ and the half-life of these antibiotics. Despite these observations, specific PK modeling could predict drug concentrations of CAZ, FEP, and MEM but not of PIP based on renal function but not based on the absolute mCL_CR_ value, suggesting several points. First, no simple upward dose adjustment can be proposed based only on mCL_CR_, a measure that is easily available to clinicians. Second, at extreme values of mCL_CR_ (e.g., >300 ml/min), there may no longer be a linear relationship between drug concentrations and mCL_CR_. Third, mCL_CR_ may not reflect precisely the true renal elimination of hydrophilic drugs such as β-lactams. The measure of CL_CR_ with 24-h urine collections informs us on the glomerular filtration rate of the kidney, but it does not account for tubular reabsorption or secretion of drugs, which may be altered in the critically ill patient (1). This may explain why even with PK modeling, mCL_CR_ could not be retained as a covariable to predict serum concentrations of PIP. Indeed, PIP has a well-described nonlinear renal clearance with a strong element of tubular secretion (31). Fourth, the measured total drug concentration of β-lactams may not allow for a good evaluation of the renal drug CL as it is the free fraction of the drug that is eliminated by the kidney (19). Fifth, other confounding factors may affect the PK of β-lactams in critically ill patients with ARC, such as changes in *V*, or nonrenal elimination of the drug (19), such as biliary clearance mechanisms, particularly in the case of PIP (32). Indeed, significant between-individual variability in values of *V* of all four antibiotics was observed in our cohort of patients.

This study has some limitations. First, this is a retrospective study performed in a single hospital. However, the data were systematically collected, and the monocentric nature of the study may increase some homogeneity in patient management. Second, the impact of antibiotic concentrations on outcome and the adequacy of increased dosage regimens after TDM were not evaluated. Third, the free fraction of β-lactams was not measured because the measurements were not routinely available in daily clinical practice. Fourth, the possible effect of significant variations in mCL_CR_ was not accounted for. However, in a retrospective study on 56 septic patients, changes in mCL_CR_ did not predict β-lactam serum concentration variations (17). Fifth, the sampling regimen was defined for TDM-based dose optimization rather than being designed for PK analysis, so the results may not be optimal for estimating PK parameters although we found that each of the drug's models performed adequately. Finally, we did not propose any new dosage regimens.

Unfortunately, our results indicate that no simple upward dose adjustment can be proposed on the basis of mCL_CR_ alone. Even if new dosage regimens can be obtained from dose simulations based on population PK models for FEP, CAZ, and MEM, they will still need to be validated in the clinical setting. In the meantime, three case reports illustrate the potential benefits of TDM-guided therapy in three septic patients with ARC where daily doses of 8 to 12 g per day of MEM were needed to cure these patients of their infections (33, 34). De Waele et al. also showed in a randomized controlled trial in critically ill patients with apparently normal renal function that TDM-guided therapy of MEM and TZP allowed for better PK/PD target attainment than non-TDM-guided therapy. Furthermore, standard dosage regimens had to be increased by up to 100% to allow patients to attain the optimal PK/PD targets (30). Without performing TDM, these extremely high dosage regimens could not be administered. Therefore, currently we recommend, when possible, TDM-guided therapy to optimize PK/PD target attainment in critically ill patients and particularly in those at risk of ARC.

## MATERIALS AND METHODS

### Study design.

In our 35-bed Department of Intensive Care at Erasme Hospital (Brussels, Belgium), daily 24-h urine collections via an indwelling catheter and daily determination of serum creatinine concentrations are performed routinely, allowing for mCL_CR_ to be calculated on a daily basis, according to the following formula: mCL_CR_ *=* (urinary creatinine × urine volume)/(serum creatinine × duration of urine collection), where serum and urinary creatinine concentrations are measured in milligrams/deciliter, urine volume is measured in milliliters, and duration of collection is in minutes. The mCL_CR_ of the day corresponds to a urine collection that was initiated at 8 a.m. on the previous day and collected for 24 h. The serum and urinary creatinine concentration values used to calculate mCL_CR_ are those measured at the end of the urine collection. Therapeutic drug monitoring (TDM) of broad-spectrum β-lactam antibiotics (FEP, CAZ, PIP, and MEM) has also been performed routinely in septic patients since October 2009.

We reviewed data from all adult patients included in an institutional database of β-lactam TDMs from October 2009 to December 2014. This database contains the following information: the date and time TDM was performed, the drug regimen administered, and the β-lactam serum concentrations obtained. Study inclusion criteria were the following: (i) diagnosis of sepsis or septic shock according to standard criteria (35), (ii) therapy with a standard dosage regimen of a broad-spectrum β-lactam (FEP/CAZ, 2 g every 8 h [q8h]; TZP, 4.5 g q6h; or MEM, 1 g q8h), and (iii) mCL_CR_ of≥120 ml/min (so that all patients close to the threshold of ARC could be included in the study) on the day of TDM. Incomplete TDMs (missing measured drug concentrations at *T*_0_ or *T*_2_) were excluded, as were TDMs performed on patients with burns or cystic fibrosis and those who were treated with extracorporeal membrane oxygenation. Patients could be included more than once. The study protocol was approved by the local Ethics Committee, who waived the need for informed consent in view of the retrospective nature of the study.

### Data collection.

We recorded demographic data, comorbidities, biological data, primary reason for ICU admission, source of infection, pathogens responsible for the infection(s), use of vasopressors, use of mechanical ventilation, number of days of β-lactam therapy, the day of TDM, and the duration of ICU stay and the 30-day ICU mortality. Severity of disease was determined with the APACHE II score (20) at ICU admission and with the SOFA score (21) on the day of TDM.

### Measurement of β-lactam serum concentrations.

β-Lactam concentrations were measured on two blood samples (3 ml each), one taken right before (*T*_0_) and the other one 2 h after (*T*_2_) the onset of a 30-min infusion. Samples were kept on ice and sent directly to the clinical chemistry laboratory; after centrifugation at 3,000 rpm at 4°C for 10 min, the supernatant was removed and analyzed. The serum concentrations of the four β-lactams were determined using high-performance liquid chromatography connected to UV spectrophotometry (HPLC-UV). Technical details have been described previously (36). For patients receiving TZP, only PIP concentrations were measured. The lower and upper limits of quantification for each analyzed β-lactam were 1 and 200 mg/liter, respectively. The coefficient of variation for all four β-lactam antibiotics was ≤7.6% for mean concentrations varying from 1 to 200 mg/liter. If a measured serum concentration was below the limits of quantification, the concentration used for PK analysis was 0.5 mg/liter.

### PK analyses.

The following equation was used to estimate the serum concentrations of the drug at one given time: ln *C_t_* = −*k_e_t* + ln *C*_0_, assuming that the steady state was reached, considering the exponential elimination of drugs, and that sampling (*T*_0_ and *T*_2_) was performed during the elimination phase. *C_t_* is the measured serum concentration at the specified time *t, C*_0_ is the virtual serum concentration at the beginning of the elimination phase, and *k_e_* is the elimination constant. The half-life (*t*_1/2_) could be calculated using the following equation: *t*_1/2_ = 0.693/*k_e_*.

The concentration-time data for FEP, CAZ, MEM, and PIP were also subject to a population pharmacokinetic analysis with the nonparametric adaptive grid (NPAG) algorithm within the freely available Pmetrics software package for R (Laboratory of Applied Pharmacokinetics and Bioinformatics, Los Angeles, CA) (37). mCL_CR_ was evaluated as a clinically relevant and physiologically plausible covariate. Covariate selection was performed using a stepwise linear regression from R and Bayesian posterior parameters. mCL_CR_ was entered into the model and statistically tested by use of the −2LL values. If inclusion of mCL_CR_ resulted in a statistically significant improvement in the −2LL values (*P* < 0.05) and/or improved the goodness-of-fit plots, then it was retained in the final model.

The goodness of fit of the model was assessed by linear regression, with an observed-predicted (both population- and individual-predicted concentrations) plot, coefficients of determination, and −2LL values. Predictive performance was based on mean prediction error (bias) and the mean bias-adjusted squared prediction error (imprecision) of the population and individual prediction models. The internal validity of the population pharmacokinetic model was assessed by the bootstrap resampling method (*n* = 1,000) and normalized prediction distribution errors (NPDEs) (38). Using the VPC method, parameters obtained from the distribution of predicted concentrations were plotted with the observed concentrations. NPDE plots were checked for normal distribution characteristics and trends in the data errors.

### Definitions for adequacy of β-lactam serum concentrations.

All TDMs were stratified into four groups according to mCL_CR_ ranges: 120 to ≤180 ml/min, 181 to ≤240 ml/min, 241 to ≤300 ml/min, and >300 ml/min. Because β-lactam antibiotics are time dependent, the PD index that best describes their efficacy is the time the unbound concentration of the antibiotic remains above the MIC of the infecting pathogen (*fT*>MIC). *In vitro* studies have shown that bactericidal effects are obtained when antibiotic concentrations remain above the MIC of the pathogen during 70%, 50%, and 40% of the dosing interval for cephalosporins, penicillins, and carbapenems, respectively (25, 26). Furthermore, maximal killing rates for β-lactam antibiotics are observed at 4× to 6× the MIC of pathogens (27). Therefore, for each TDM, the drug concentration at the end of the optimal period of time (CT) was calculated, corresponding to 70% of the dose interval for FEP/CAZ, 50% for PIP, and 40% for MEM. A CT of ≤4× MIC was defined as an insufficient serum concentration, and a CT of >4× MIC was defined as adequate. We used clinical breakpoints for Pseudomonas aeruginosa, as defined by the EUCAST as our target MICs: 8 mg/liter for FEP/CAZ, 16 mg/liter for PIP, and 2 mg/liter for MEM (39). Adequate CTs for treating infections due to Pseudomonas aeruginosa were therefore >32 mg/liter for FEP/CAZ, >64 mg/liter for PIP, and >8 mg/liter for MEM.

As a *post hoc* analysis, we also analyzed adequacy of serum concentrations to treat infections due to Enterobacteriaceae spp. because these were the most frequently documented pathogens in our study. We once again used clinical breakpoints for Enterobacteriaceae spp., as defined by EUCAST: 1 mg/liter for FEP/CAZ, 8 mg/liter for PIP, and 2 mg/liter for MEM (39). Adequate CTs for treating infections due to Enterobacteriaceae spp. were therefore >4 mg/liter for FEP/CAZ, >32 mg/liter for PIP, and >8 mg/liter for MEM.

### Statistical analysis.

Statistical analyses were performed using the software package SPSS, version 24.0, for Windows NT (SPSS, Inc., Chicago, IL, USA). Descriptive statistics were computed for all study variables; discrete variables were expressed as counts (percentage), and continuous variables were expressed as means ± standard deviations or medians (interquartile range [IQR]). Categorical data were compared using a chi-square test or Fisher's exact test, as appropriate, and continuous variables were compared using a Mann-Whitney U test. We looked for a relationship between mCL_CR_ and drug concentrations at different time points (*T*_0_, *T*_2_, and the predefined optimal periods of time for each β-lactam), between mCL_CR_ and *T* >4× MIC, between mCL_CR_ and CL, and between mCL_CR_ and half-life for all drugs. The Spearman's correlation coefficient (*r*) was used to determine linear correlation as appropriate. Association between variables was tested by simple regression analysis and the coefficient of determination (*R*^2^) in case of nonlinear correlation. All tests were two-tailed, and a *P* value of <0.05 was considered statistically significant.

## Supplementary Material

Supplemental file 1
