# Supplementary material for: β-Lactam Dosage Regimens in Septic Patients with Augmented Renal Clearance
Source: Antimicrob Agents Chemother. 2018 Aug 27;62(9):e02534-17. doi: 10.1128/AAC.02534-17 (PMC6125556; doi:10.1128/AAC.02534-17)

Supplementary Figure 1S. Goodness-of-fit plot of the final models with r^2 correlations between observed and predicted concentrations (both individual and population) for: 1SA) Cefepime, **1SB)** Ceftazidime, **1SC)** Piperacillin, **1SD)** Meropenem.

A) Cefepime

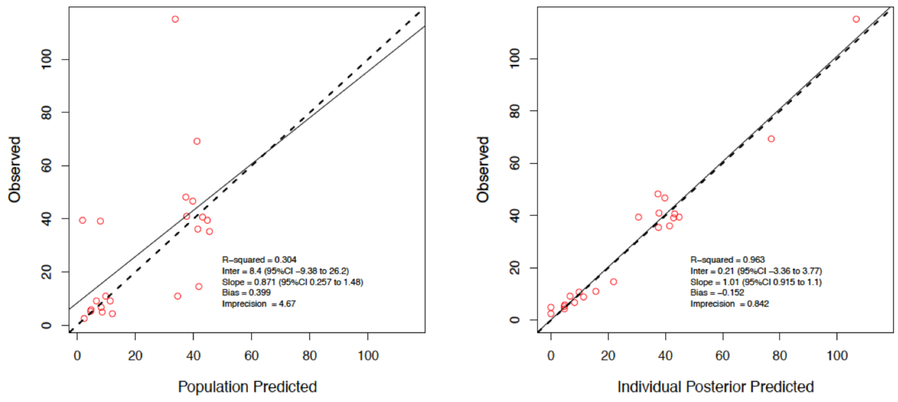

B) Ceftazidime

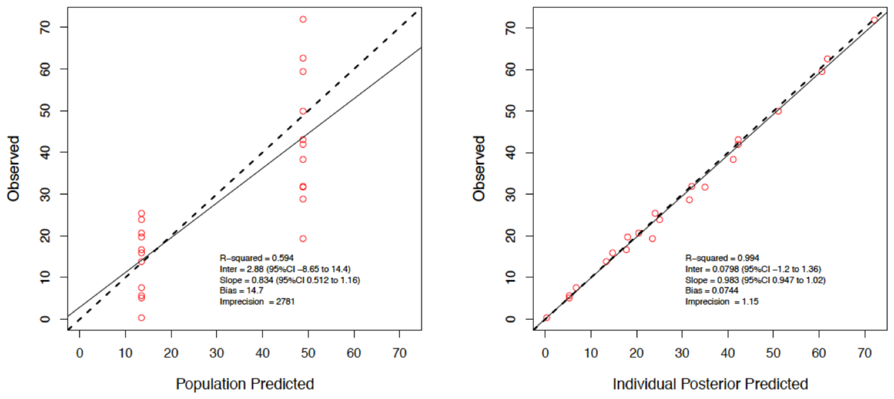

C) Piperacillin

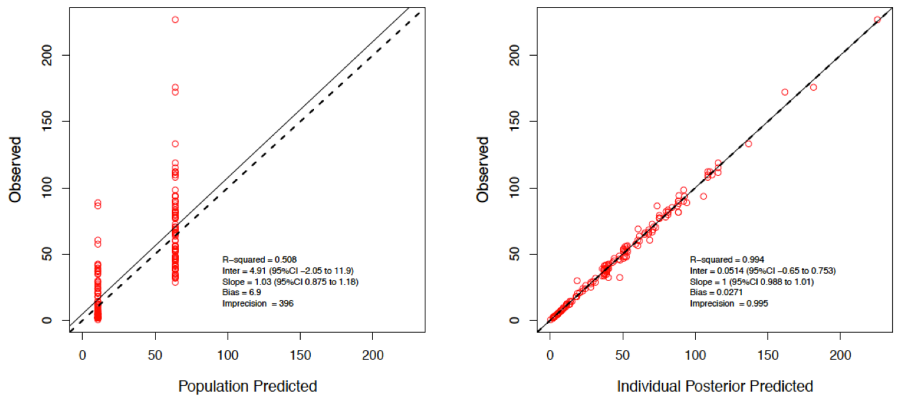

D) Meropenem

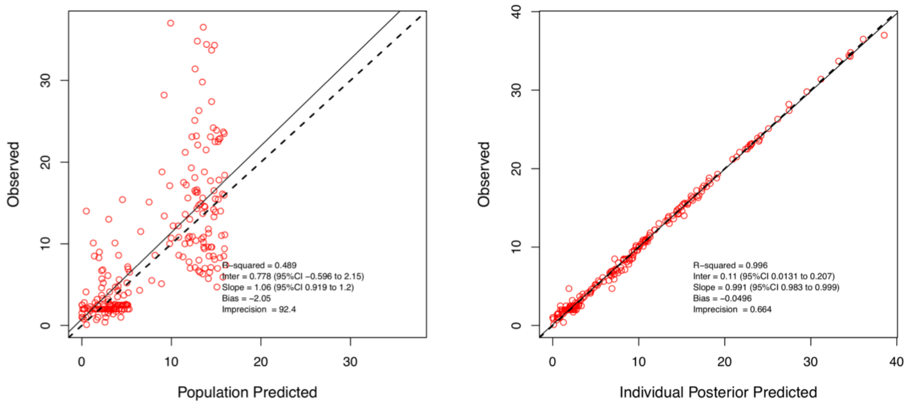

Supplement: Supplemental file 1 [file zac009187455s1.pdf]
